# Hypoglycemia Among Type 1 Diabetes Patients After Insulin Use in Southwest Ethiopia

**DOI:** 10.3389/fendo.2021.684570

**Published:** 2021-10-08

**Authors:** Tewodros Yosef

**Affiliations:** Department of Epidemiology and Biostatistics, School of Public Health, College of Medicine and Health Sciences, Mizan-Tepi University, Mizan Teferi, Ethiopia

**Keywords:** hypoglycemia, diabetes, insulin use, Ethiopia, Metu Karl referral hospital

## Abstract

**Introduction:**

Glycemic control is a valuable goal for people with diabetes; however, the greatest challenge to achieving tight glycemic control is hypoglycemia. Hypoglycemic events are probably common in type 1 diabetes; however, little is known about hypoglycemia in Ethiopia. Therefore, this study aimed to assess the prevalence and the associated factors of hypoglycemia among type 1 diabetes (T1D) patients after insulin use at Metu Karl Referral Hospital in southwest Ethiopia.

**Materials and Methods:**

A hospital-based cross-sectional study was conducted among 242 T1D patients at Metu Karl Referral Hospital in southwest Ethiopia. The prevalence of hypoglycemia was assessed by a structured questionnaire through a face-to-face interview in which all the possible symptoms of hypoglycemia were included. If the patients reported that they had experienced the symptoms at least two times in a month and the symptoms were relieved upon consuming sugar/candy/honey, such cases were considered to have had a hypoglycemic episode. Binary logistic regression analysis was done to identify the factors associated with the occurrence of hypoglycemia.

**Results:**

Out of 242 T1D patients interviewed, 114 (47.1%) had self-reported hypoglycemia. The most reported symptom of hypoglycemia was sweating (91.7%), followed by dizziness and hunger and nausea with a prevalence of 24.8 and 14.5%, respectively. The study also found that educational level with reading and writing skills up to primary level [adjusted odds ratio, AOR = 0.41; 95% confidence interval, CI (0.19–0.88)] and secondary level and above [AOR = 0.32, 95% CI (0.14–0.70)], poor knowledge of diabetes [AOR = 2.26, 95% CI (1.06–4.84)], good knowledge of insulin self-administration [AOR = 0.54, 95% CI (0.30–0.99)], and duration of insulin use ≥5 years [AOR = 3.93, 95% CI (1.44–10.7)] were factors associated with hypoglycemia.

**Conclusions:**

The prevalence of hypoglycemia was found remarkable. We can conclude that hypoglycemia is of public health importance among T1D patients. Since the study assesses hypoglycemia after insulin injection, this prevalence may be due to the poor practice of insulin injection. Therefore, imparting education on the proper technique of insulin administration should be considered at each follow-up visit.

## Introduction

Diabetes mellitus significantly contributes to the global health burden in the 21^st^ century (1). Achieving good glycemic control is a valuable goal for people with diabetes (2); however, hypoglycemia is the greatest challenge to achieving tight glycemic control (3–7), which results in declined drug compliance, cardiovascular events, and even mortality (3). It is also related to a negative impact on health-related quality of life, healthcare resource use, and work productivity (5).

Hypoglycemic events are probably common in diabetic patients who use insulin (4, 8–12), and patients with type 1 diabetes (T1D) are more likely to develop hypoglycemia as compared with type 2 diabetic patients (4, 5, 13, 14). One of the most feared complications of diabetes treatment is hypoglycemia (15), which commonly occurs in clinical practice as approximately 90% of all patients who receive insulin have experienced hypoglycemic episodes (16).

Hypoglycemia, also known as an insulin reaction or insulin shock, is a clinical and biological syndrome caused by an abnormal decrease in plasma glucose levels to below 70 mg/dl and responsible for non-specific signs and symptoms, including shakiness, nervousness, sweating, chills and clamminess, dizziness, hunger and nausea, confusion, weakness, sleepiness, seizures, and loss of consciousness (17, 18). It is categorized as either mild or severe based on the seriousness of the event and whether it requires external support or is self-limiting (19, 20).

The factors associated with hypoglycemia are varied. It may include age, sex, occupational status, residence, body mass index, missed meals or inadequate caloric intake, alcohol consumption, concurrent use of an opioid, level of fasting blood sugar, blood glucose monitoring, glucose checkup period, insulin or diabetes medications, duration of diabetes, and presence of stroke (3, 5, 8, 21–24).

Individuals with T1D require a lifelong insulin supply for good treatment results. The most common problem among patients with T1D is lack of adherence to insulin therapy. The fear of hypoglycemia is the principal factor associated with their nonadherence to insulin therapy (25, 26). Like other diabetes complications, prevention is the best remedy for hypoglycemia, and recognizing the associated risk factors is the first step (27). Recognition of the problem, assessment of the risk factors, and application of the principles of intensive glycemic management are very important in reducing the possibility of hypoglycemia (28). The prevention and treatment of hypoglycemia in patients with T1D needs greater vigilance and education (29). Hypoglycemia in people living with diabetes mellitus is an unexplored area in literature in Africa (30), including Ethiopia. Any intervention aimed at preventing hypoglycemia in patients with T1D can only be possible when there is sufficient data on the issue. Therefore, this study aimed to assess the prevalence and the associated factors of hypoglycemia among T1D patients after insulin use at Metu Karl Referral Hospital in southwest Ethiopia.

## Materials and Methods

### Study Setting and Period

The study was conducted at Metu Karl Heinz Referral Hospital (MKRH) from January 1 to 30, 2019. MKRH is located in the Oromia region, Ilu Abbabor zone, Metu town, 600 km southwest of Addis Ababa, the capital city of Ethiopia. The details of the study area were better described in a previous publication (31).

### Study Design and Populations

A cross-sectional study was carried out. All insulin-dependent self-injecting T1D patients who had chronic follow-up visits at Metu Karl Referral Hospital during the study period were included in the source population. The study population was randomly selected among self-injecting T1D patients who fulfill the inclusion criteria during the study period. The details of the study population were better explained in a previous publication (31).

### Sample Size Determination

The sample size was determined using a single-population proportion formula with an input of the expected proportion of hypoglycemia patients (50%), 5% margin of error, and 95% confidence level. The sample size computed was 384. However, the source population (*N* = 535 patients who were taking insulin therapy at the time of data collection) was less than 10,000; by applying the correction formula, it then became 223, but by adding 10% for non-response compensation, the final sample size was determined to be 245.

### Sampling Method

Unless there is any disease-related emergency, every diabetes patient is set with a monthly appointment to have a checkup and for them to collect their monthly medication. A systematic random sampling method was used to select the study populations. The details of the sampling method were better explained in a previous publication (31).

### Study Variables and Measurements

The dependent variable was hypoglycemia. The independent variables were sociodemographic factors (age, sex, marital status, and educational status), knowledge of diabetes, knowledge of insulin self-administration, and health profiles [family history of diabetes mellitus (DM), membership in a DM association, and duration of insulin use].

Hypoglycemia is a clinical and biological syndrome which is responsible for non-specific signs and symptoms, including shakiness, nervousness, sweating, chills and clamminess, dizziness, hunger and nausea, confusion, weakness, sleepiness, seizures, and loss of consciousness (18), and the symptoms are relieved upon consuming sugar/candy/honey (32).

Prevalence was defined as the frequency at which the study subjects experienced at least two symptoms of hypoglycemia in the last month.

Good knowledge of insulin self-administration refers to a person who scores greater than the mean value (≥5 or ≥62.5%) of knowledge-based questions; otherwise, knowledge of insulin self-administration is considered poor (31).

Awareness of hypoglycemia is demonstrated by participants who answered “always” to the question “Can you feel when your blood sugar is low?”; otherwise, they were classified as unaware of hypoglycemia (33).

### Data Collection Instrument and Procedures

The data were collected through a face-to-face interview. The prevalence of hypoglycemia was assessed by a structured questionnaire in which all the possible symptoms of hypoglycemia were included (18). Two criteria were used, both of which suggest that the symptoms of a patient result from hypoglycemia. The criteria include the following:

1. symptoms of hypoglycemia happening after injecting insulin
2. relief of the symptoms using sugar/candy/honey

The common symptoms of hypoglycemia were listed. The patients were asked if they had ever experienced any one of the listed symptoms after injecting insulin in the past month. If the answer was “yes”, then they were enquired about the frequency of the above-mentioned symptoms. If they answered that they had experienced the symptoms at least two times in a month and the symptoms were relieved upon consuming sugar/candy/honey, then they were considered to have had a hypoglycemic episode. Hence, if the abovementioned criteria were met, the patients were identified to have had a hypoglycemic episode. A hypoglycemic event that required the assistance of another person or which required medical assistance in a hospital for corrective measures was documented as a severe episode of hypoglycemia. The questionnaire was tested for reliability and validity. The face validity was performed by an internist who worked at the diabetes clinic of the hospital. The reliability of the analysis was determined using Cronbach’s alpha test where the reliability coefficient was found to be significant (Cronbach’s alpha: 0.77). To assess the quality, the questionnaire had been pre-tested in similar setups before the actual data collection was commenced. Training was given to data collectors and supervisors concerning the objective and the process of data collection.

### Data Processing and Analysis

The data collected were entered into Epi-data, version 4.2.0.0, and analyzed using SPSS, version 20. Binary logistic regression analysis was done. Independent variables with a *P*-value of less than 0.25 in bivariate logistic regression were included in multivariable logistic regression. Multivariable logistic regression analysis was done to control for potential confounding factors and identify the most important determinate variables. The level of significance was declared at *P*-value <0.05 in the multivariable logistic regression analysis.

## Results

### Socio-Demographic Characteristics

Of the sample size of 245, 242 T1D patients have participated in the study, yielding a response rate of 98.7%. The mean age of the respondents was 33.7 ( ± 12.6 SD) years, with a range of 19 to 70 years. The majority (45.4%) of the respondents were in the age group of 19–28 years. One hundred eight (44.6%) of the participants were Protestant followers. One hundred twelve (46.3%) and 58 (24%) of the respondents were out of marriage and cannot read and write, respectively (**Table 1**).

**Table 1:** Socio-demographic characteristics of type 1 diabetes patients at metu karl heinz referral hospital in ethiopia.

Variables	Categories	Frequency	Percent
Age group (years)	19–28	110	45.4
	29–38	57	23.6
	39–48	41	16.9
	49–58	14	5.8
	59–70	20	8.3
Sex	Male	98	40.5
	Female	144	59.5
Religion	Protestant	108	44.6
	Orthodox	75	31.0
	Muslim	59	24.4
Marital status	Out of marriage	112	46.3
	Within marriage	130	53.7
Educational status	Cannot read and write	58	24.0
	Can read and write up to G-8	85	35.1
	Secondary and above	99	40.9

### Health-Related Profiles

Ninety-nine (40.9%) of the respondents had a family history of diabetes. One hundred forty-one (58.3%) of the participants were members of Ethiopian diabetes associations. One hundred ninety-five (80.6%) of the respondents had good knowledge of diabetes. The majority (61.6%) of the respondents had poor knowledge of insulin self-administration. More than three-fourth (88%) of the respondents were treated for five or more years (**Table 2**).

**Table 2:** Health-related profiles of type 1 diabetes patients at metu karl heinz referral hospital in ethiopia.

Variables	Categories	Frequency	Percent
Family history of diabetes	Yes	99	40.9
	No	143	59.1
Member of the diabetes association	Yes	141	58.3
	No	101	41.7
Knowledge of diabetes	Good	195	80.6
	Poor	47	19.4
Knowledge of insulin self-administration	Good	93	38.4
	Poor	149	61.6
Duration of insulin therapy (years)	<3	11	4.5
	3–5	18	7.4
	≥5	213	88.0

### Prevalence of Self-Reported Hypoglycemia

Of the 242 respondents interviewed, 170 (70.3%) respondents were aware of hypoglycemia symptoms. One hundred fourteen (47.1%) respondents reported a history of hypoglycemia after injecting insulin. A total of 366 hypoglycemic events happened in the last month. The most reported symptom of hypoglycemia was sweating (91.7%), followed by dizziness and hunger and nausea with a prevalence of 24.8 and 14.5%, respectively (**Table 3**). Of those who developed hypoglycemia, 86 (75.4%), 20 (17.6%), and eight (7.0%) were managed by home treatment using sugar, candy, and honey, respectively. No respondent reported inpatient admission to the hospital due to hypoglycemia.

**Table 3:** Self-reported symptoms of hypoglycemia among type 1 diabetes patients at metu karl heinz referral hospital in ethiopia.

Symptoms of hypoglycemia	Categories	Frequency (*n*)	Percent (%)
Sweating	Yes	222	91.7
	No	20	8.3
Dizziness	Yes	60	24.8
	No	182	75.2
Hunger and nausea	Yes	35	14.5
	No	207	85.5
Sleepiness	Yes	22	9.1
	No	220	90.9
Chills and clamminess	Yes	18	7.4
	No	224	92.6
Shakiness	Yes	9	3.7
	No	233	96.3

### Factors Associated With Hypoglycemia

After adjusting for age group, educational status, and knowledge of insulin self-administration as confounding factors, educational status, knowledge of diabetes and insulin self-administration, and duration of insulin use were significantly associated with hypoglycemia at a *P*-value <0.05 (**Table 4**).

**Table 4:** Factors associated with hypoglycemia among type 1 diabetes patients at metu karl heinz referral hospital in ethiopia.

Variables	Categories	Hypoglycemia		COR (95% CI)	AOR (95% CI)
		Yes	No		
**Age group (years)**	19–28	50	60	1	1
	29–38	25	32	0.94 (0.49–1.79)	1.10 (0.53–2.28)
	39–48	23	18	1.53 (0.75–3.16)*	1.00 (0.42–2.35)
	49–58	5	9	0.67 (0.21–2.12)	0.36 (0.10–1.26)
	59–70	11	9	1.47 (0.56–3.82)	1.15 (0.40–3.32)
**Educational status**	Cannot read and write	35	23	1	1
	Can read and write up to G-8	37	48	0.51 (0.26–0.99)**	0.41 (0.19–0.88)**
	Secondary and above	42	57	0.48 (0.25–0.94)**	0.32 (0.14–0.70)**
**Knowledge of diabetes**	Yes	85	110	1	1
	No	29	18	2.09 (1.09–4.00)**	2.26 (1.06–4.84)**
**Knowledge of insulin self-administration**	Good	64	85	0.65 (0.39–1.09)*	0.54 (0.30–0.99)**
	Poor	50	43	1	1
**Duration of insulin use**	<5 years	6	23	1	1
	≥5 years	108	105	3.94 (1.54–10.1)**	3.93 (1.44–10.7)**

AOR, adjusted odds ratio; CI, confidence interval; COR, crude odds ratio.

*p-value < 0.25, **p-value < 0.05.

## Discussion

Indeed it is impossible to eliminate hypoglycemia from the lives of T1D patients (34), but recognition of the problem, evaluation of the risk factors, and application of the principles of intensive glycemic management are very important for minimizing hypoglycemia (28). Based on the abovementioned facts, this study aimed to assess the prevalence and the associated factors of hypoglycemia among T1D patients after insulin use at Metu Karl Referral Hospital in southwest Ethiopia. As a result, the proportion of self-reported hypoglycemia among type 1 diabetes was 114 (47.1%), at 95% confidence interval of 40.8–53.4%. This study was in line with 50% of the RECAP-DM study in Argentina (35). This finding was lower than 86.7% in Debre Markos Referral Hospital (5), 88% in Tikur Anbessa Specialized Hospital (23), and 94.3% in St. Paul’s Hospital Millennium Medical College (22) studies in Ethiopia and 91.7% from HAT study in Brazil (36), 97.1% in Colombia (37), 97.4% from an international survey in nine countries (38), and 100% from the Southeast Asia cohort of IO HAT study (39). The variation observed between this and other studies may be due to the operational definition used. Unlike other studies, this study classified the individuals as having experienced hypoglycemia when they experience the hypoglycemia symptoms at least two times in 1 month. Besides that, unlike this study, the other studies with a reported high prevalence were done in more urban and vigilant societies, such that the subjects may be easily aware of a hypoglycemic episode, resulting in increased reports of hypoglycemia symptoms.

Respondents who can read and write and with up to primary and secondary and above education were 59 and 68%, respectively, less likely to develop hypoglycemia. Being educated was significantly associated with less occurrence of hypoglycemia. This could be because an individual who had better education may be associated with better knowledge of good insulin administration techniques to avoid the problem resulting from using an inappropriately high dose of insulin. This finding was supported by Wako et al., who revealed that low educational status was associated with the occurrence of hypoglycemia (23).

Respondents who had good knowledge of insulin self-administration were 46% less likely to develop hypoglycemia than those who had poor knowledge of insulin self-administration. Having good knowledge may be related to less chance of acquiring hypoglycemia. This could be explained by the good knowledge of insulin self-administration, which may prevent related hypoglycemia due to an inappropriately high dose of insulin.

Respondents who had poor knowledge of diabetes had 2.3 times increased odds of developing hypoglycemia than those who had good knowledge of diabetes. Having good knowledge of diabetes was significantly associated with less chance of developing hypoglycemia. This could be due to the good knowledge of diabetes being related to good knowledge of managing diabetes and the ways on how to prevent related complications. This finding was in line with a study by Gebrewahd and Teklewoini, which revealed that good knowledge of diabetes and hypoglycemia and a favorable attitude towards diabetes are positive predictors of good hypoglycemia prevention practice (40).

Respondents who were treated for 5 or more years had 3.9 times increased odds of developing hypoglycemia than those who were treated for less than 5 years. One of the reasons for recurrent hypoglycemia in patients with diabetes for more than 5 years could be “unrecognized” chronic kidney disease, as the kidney is the site for the degradation of insulin. Besides this, it could be due to the fact that those who are treated for a long duration may have become fatigued and caught napping on the techniques to administer insulin by themselves. This finding was supported by studies done in Ethiopia (5, 22).

### Strengths and Limitations of the Study

Despite that this study assesses the most understudied problem in Ethiopia, it has some limitations. First was the failure to use a finger stick blood glucose test since hypoglycemia was based on self-reported hypoglycemia symptoms. Secondly, the study was carried out in a single referral hospital; the findings from this study cannot be generalized to different populations and settings across Ethiopia. Lastly, there was failure to assess the daily activity of patients and their nutritional intake as factors associated with the outcome variable.

## Conclusion

The prevalence of hypoglycemia among T1D patients in the study area was found remarkable. We can conclude that hypoglycemia is a public health problem among T1D patients. Since the study assesses hypoglycemia after insulin injection, this prevalence may be due to the poor practice of insulin injection. Therefore, imparting education on the proper technique of insulin administration should be considered at each follow-up visit.

## Data Availability Statement

The original contributions presented in the study are included in the article/Supplementary Material. Further inquiries can be directed to the corresponding author.

## Ethics Statement

Ethical approval was obtained from Mizan-Tepi University Ethical Review Board. The ethical approval number was MTUERB/86/2019. Permission was obtained from Metu Karl Referral Hospital. All study participants were informed about the purpose of the study, their right to deny participation, anonymity, and confidentiality of the information. Written informed consent was also obtained before participation in the study.

## Author Contributions

The author confirms being the sole contributor of this work and has approved it for publication.

## Conflict of Interest

The author declares that the research was conducted in the absence of any commercial or financial relationships that could be construed as a potential conflict of interest.

## Publisher’s Note

All claims expressed in this article are solely those of the authors and do not necessarily represent those of their affiliated organizations, or those of the publisher, the editors and the reviewers. Any product that may be evaluated in this article, or claim that may be made by its manufacturer, is not guaranteed or endorsed by the publisher.

## Abbreviations

AOR, adjusted odds ratio; CI, confidence interval; COR, crude odds ratio; DM, diabetes mellitus; G-8, grade 8; MKRH, Metu Karl Referral Hospital; SPSS, Statistical Package for the Social Sciences; SD, standard deviation; T1D, type 1 diabetes.
